# Local adaptation and host specificity to copepod intermediate hosts by the tapeworm *Schistocephalus solidus*

**DOI:** 10.1002/ece3.10155

**Published:** 2023-06-06

**Authors:** Kum C. Shim, Christopher R. Peterson, Daniel I. Bolnick

**Affiliations:** ^1^ Department of Integrative Biology University of Texas at Austin Austin Texas USA; ^2^ Department of Ecology and Evolutionary Biology University of Connecticut Storrs Connecticut USA

**Keywords:** coevolution, copepods, infection, local adaptation, stickleback

## Abstract

Host–parasite coevolution may lead to patterns of local adaptation in either the host or parasite. For parasites with complex multi‐host life cycles, this coevolution may be more challenging as they must adapt to multiple geographically varying hosts. The tapeworm *Schistocephalus solidus* exhibits some local adaptation to its second intermediate host, threespine stickleback, to which the parasite is strictly specialized. However, the tapeworm's adaptation to its first intermediate host (any of a number of copepod species) is not documented. We investigated if there was local adaptation and host specify in the tapeworm *Schistocephalus solidus* to its copepod first intermediate hosts. We exposed copepods from five lakes in Vancouver Island (BC, Canada) to local (i.e. same lake) and foreign tapeworms in a reciprocal exposure experiment. Results indicate that the tapeworm is not locally adapted to the copepods. Instead, we observed moderate‐effect host specificity, infection rates being higher in certain copepod species than in others. Infection rates also varied among cestode populations. These results show that although *S. solidus* infects multiple copepod genera, they are not equally competent hosts. Differences in *S. solidus* epidemiology among lakes is likely to be driven more by this partial specialization, than by local adaptation to first intermediate hosts.

## Graphical abstract

We test for local adaptation between a parasitic tapeworm and its first intermediate hosts, cyclopoid copepods. We find evidence of moderate host specialization on one of two copepod genera, but no local adaptation.

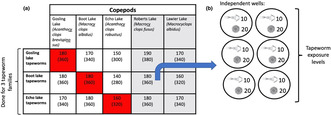

## 1 INTRODUCTION

One of the most intriguing features of parasites with complex life cycles is their ability to infect several very disparate hosts during each of their life stages (Schmid‐Hempel 2011). Transmission of these parasites (especially helminths) usually involves search for, and penetration of, their intermediate hosts. These parasites then infect their final hosts when the intermediate hosts are predated, a process which they may facilitate by manipulating intermediate host vulnerability. Thus, complex‐life‐cycle parasites usually lower the overall fitness of their intermediate hosts (i.e. increased predation). Such parasites cannot readily control what apex predator eats their intermediate host, and so they cannot be as selective on infecting final hosts (Noble et al., 1989; Poulin, 2007; Schmid‐Hempel, 2011). Accordingly, these parasites should be more host‐specific to intermediate than to final hosts (Noble et al., 1989, Poulin, 2007), as a form of bet‐hedging against the range of possible final predators that might consume them. Increased host specificity and negative fitness effects imply that host–parasite coevolution and local adaptation may be more likely between parasites and their intermediate hosts (Lively et al., 2004).

Moreover, in host–parasite coevolution, the species with the higher dispersal rates is predicted to locally adapt to the other (Gandon & Nuismer, 2009; Greischar & Koskella, 2007; Morgan et al., 2005). This theoretical result is contrary to our usual expectation that dispersal and gene flow homogenize populations and counter‐act divergent selection (Lenormand, 2002). But in antagonistically interacting species, gene flow (in moderation) provides genetic diversity that aids in adapting to the opposing species (Gandon & Nuismer, 2009). Parasite dispersal rates are usually higher than their intermediate hosts' dispersal (Hoeksema & Forde, 2008; Mazé‐Guilmo et al., 2016), so parasites should be more locally adapted to their intermediate hosts than vice‐versa. Local adaptation can also be facilitated by parasites' high dispersal rates which counter‐intuitively gives an advantage during antagonistic coevolution with hosts (Hoeksema & Forde, 2008, Mazé‐Guilmo et al., 2016), and by their capacity for facultative hermaphroditic reproduction.

Combining all the propositions above (i.e., hosts‐specificity, negative fitness effects on their intermediate hosts, and higher dispersal rates), parasites with complex life cycles, especially those that are hermaphroditic, should be often locally adapted to their intermediate hosts. However, many parasites have multiple intermediate hosts. These may be sequential hosts which the parasite passes through via two successive predation events. For instance, the cestode *Schistocephalus solidus* (Eucestoda: Pseudophyllidea) first infects cyclopoid copepods, which are eaten by threespine stickleback (*Gasterosteus aculeatus*), which are ultimately eaten by piscivorous birds (Barber & Scharsack, 2009; Dubinina, 1980). Or, the parasite may infect any of several alternative host species at a given trophic level (*S. solidus* infects multiple copepod genera). Do such multi‐host parasites exhibit local adaptation at both first and second intermediate hosts? Do they locally adapt to each of several alternative hosts?

To answer such questions, we tested for local adaptation using the hermaphroditic tapeworm *Schistocephalus solidus* and its first intermediate hosts, freshwater cyclopoid copepods. This tapeworm is found mainly in Holarctic lakes where (as noted above) it infects cyclopoid copepods, stickleback, and finally warm‐blooded vertebrates (usually birds). The tapeworm reproduces sexually in the finals hosts' intestines and its eggs are dispersed with these hosts' feces, so the tapeworm has higher dispersal rates than its first two intermediate hosts which rarely disperse between even adjacent lakes (Caldera & Bolnick, 2008). The tapeworm can be bred in‐vitro, making it an excellent laboratory system for host–parasite studies (Barber, 2013; Barber & Scharsack, 2009; Smyth, 1990). The tapeworm is not host specific to its final hosts, infecting several species of birds and even fish‐eating mammals like otters (Dubinina, 1980; Hoberg et al., 1997). However, the tapeworm is strictly host‐specific to the threespine stickleback (Barber, 2013; Dubinina, 1980), failing to infect even other stickleback species (which have their own *Schistocephalus* species). The tapeworm affects negatively the fitness of the fish (Weber et al., 2022; Weber, Steinel, et al., 2017), and is locally adapted to this host (Hafer, 2017; Kalbe et al., 2016). In laboratory infections, this tapeworm had negative fitness consequences to lab‐reared *Macrocyclops albidus* copepods (Benesh, 2010; Wedekind, 1997); however, no work has been done on wild copepod species that are sympatric with the tapeworm to establish host‐specificity and local adaptation, as has been done with stickleback.

We hypothesized that this tapeworm might be locally adapted to their copepod hosts (as in their stickleback host), or might specialize on particular copepod species regardless of origin, or both. To test these hypotheses, we conducted reciprocal infection trials using factorial combinations of *S. solidus* tapeworms and native copepod species collected from lakes on Vancouver Island. We tested for local adaptation by estimating infection rates in copepods by local (same lake) and foreign (different lake) tapeworms, as well as estimating infection intensity (number of parasites inside hosts) when copepods were successfully infected. Infection rates and intensities can be related or may be controlled by distinct immune processes so evaluating both provides a more complete picture of infection success. To measure host specificity, we infected different copepod genera (from different lakes) with the tapeworm and measured infection success in each genus. Results indicate that there was no local adaptation by the tapeworm in the copepods, but there was host specificity as a specific crustacean genus had overall higher infection rates than another used in this experiment.

## 2 MATERIALS AND METHODS

### 2.1 Copepod colonies

We used copepods from established laboratory colonies from five lakes on Vancouver Island (Boot, Echo, Gosling, Lawier, and Roberts Lakes; the coordinates for these lakes are in Table S1). These colonies were established from plankton tows collected on September 15, 2017, and June 24, 2018. Colonies were kept in five gallon buckets at 20°C and under 16:8 h light: dark to simulate summer conditions in Vancouver Island until the start of the experiment on October 20, 2018. Maintaining the copepods for up to a year allows us to minimize maternal effects and other forms of trans‐generational plasticity.

We fed copepods in each bucket weekly with ~500 mL of *Paramecium caudatum* and mixed rotifer cultures plus a ground protozoan pellet, both from Carolina Biological Supply Company. We also added 10–20 autoclaved wheat seeds once a month to each bucket for bacterial growth, which contributed to the copepod and paramecium diets. Before the start of the experiment, we identified each lake's copepods to species level under a dissecting scope and using the Image‐Based Key to the Zooplankton of North America (Aliberti et al., 2013). The laboratory colonies for each lake only had one surviving copepod species just before the start of the experiment. These were *Macrocyclops albidus* for Boot and Lawier Lakes, *Macrocyclops fuscus* for Roberts Lake, *Acanthocyclops robustus* for Echo Lake, and *Acanthocyclops brevispinosus* for Gosling Lake. All these copepods were from the order Cyclopoida.

### 2.2 Tapeworm colonies

We used tapeworm eggs from three lakes in Vancouver Island (Boot, Echo, and Gosling Lakes). The two additional lakes providing copepods (Lawier and Roberts Lakes) do not support native *S. solidus* populations (the stickleback are absent [Lawier], or uninfected [Roberts]). Thus, we infected copepods from five lakes with tapeworms from three. The advantage of this design is that copepods from Lawier and Roberts lakes could be highly susceptible to the tapeworm due to their lesser exposure to the parasites; thus, serving as naturally parasite‐free controls probably not affected by coevolution with the parasite. The tapeworm eggs were collected from laboratory crosses of randomly chosen wild tapeworms obtained from infected fish, following established methods (Smyth, 1990; Weber, Steinel, et al., 2017). These crosses were done in June – Sept. 2018, and the eggs were kept at 4°C until the experiment.

### 2.3 Experimental set‐up

**FIGURE 1:**
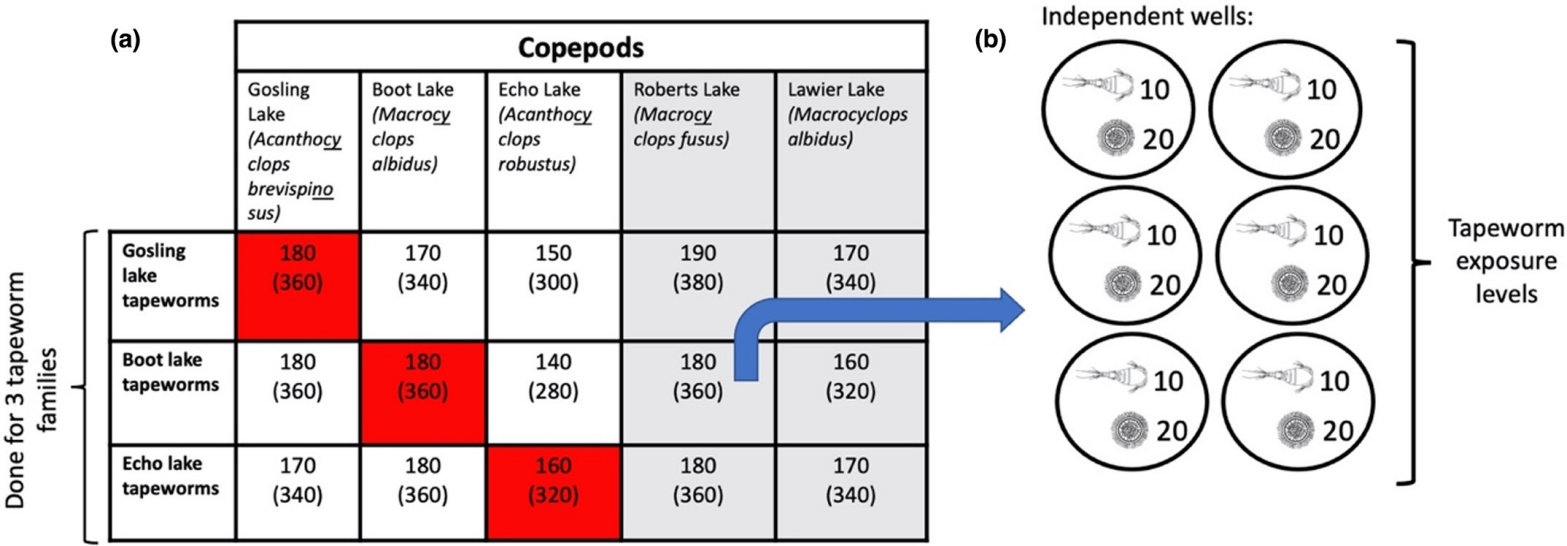
Graphical representation of the experiment setup. (a) The combinations of the tapeworm *Schistocephalus solidus* by copepod exposures, using three tapeworm families per lake; red squares indicate tapeworms exposed to sympatric copepods. Roberts and Lawier Lakes are shaded in gray representing control lakes where the tapeworm is lacking in threespine sticklebacks. The numbers inside each square represent total numbers of copepods and tapeworms used (the latter in parenthesis). Names of the copepod species used are below each lake's names. (b) A diagram of how each tapeworm family was exposed to each lake's copepods (in this example Boot Lake tapeworms to Roberts Lake copepods): in six different wells from different 6‐well plates, each with 10 copepods exposed to 20 tapeworm larvae. All well positions for all exposures in panel A were randomized in the 6‐well plates, and the position for each 6‐well plates were also randomized in the experimental room.

To test for tapeworm local adaptation and host specificity to copepods, we carried out a reciprocal infection experiment (Figure 1), exposing the copepods from each lake to local and foreign tapeworm larvae (coracidia) from three lakes (i.e., Boot, Echo, and Gosling lakes). We hatched tapeworm eggs and exposed the coracidia to copepods following published methods (Smyth, 1990, Weber, Steinel, et al., 2017). We used six‐well plates, each well holding a different combination of copepods (*n* = 10 individuals per well) from a lake and tapeworms (*n* = 20 coracidia per well) from the same or a different lake (Figure 1). We used a combination of 1:2 copepod to tapeworm ratio to account for the short lifespan (~24 h) of the parasite (Dubinina, 1980). We used three tapeworm families or strains per lake. We also had six to eight wells per lake with copepods unexposed to tapeworms as negative controls to measure tapeworm exposure and infection effects on host mortality (Table S2). The plates were kept in the same conditions as the copepod colonies (i.e. 20°C and 16:8 h light: dark). We randomized the positions of the copepod‐tapeworm combinations within plates, and plate locations within the incubator. We dissected each surviving copepod to ascertain infection status 17–22 days post exposure when tapeworms reached maximum size inside copepods (Dubinina, 1980).

In total, we used 49 6‐well plates, exposing 2890 copepods (10 per well) with 5780 tapeworms (20 per well, nine families in total, three per lake. See Table S2). At the end of experiment, 1622 exposed and 330 control copepods survived. The survival rate for copepods in the experiment was 56%. Exposure to tapeworms did not affect copepod survival (*p* value = .996, Figure S2).

### 2.4 Bayesian data analysis

We used mixed‐effect hurdle models to simultaneously estimate the effect of copepod and parasite origin on infection rate (prevalence) and intensity (number of worms per successfully infected copepod). Conceptually, these models combine a logistic regression on parasite presence/absence with a truncated Poisson regression on non‐zero parasite counts. Our models considered tapeworm lake and its interaction with either copepod genus or lake as fixed effects. Note that each lake provided only one genus but genera were replicated in at least two lakes each (Acanthocyclops sp. from two lakes, *Macrocyclops* sp. from three lakes). We also included an indicator for whether the tapeworm and copepod were from the same lake (i.e. “native”). Plate number and tapeworm lake were included as random effects. The full model contains all of these terms as predictors for both prevalence and incidence. We created a series of reduced models from a list of all possible combinations of predictors, excluding models that contained interactions without their main effects, copepod lake without genus, and tapeworm family without lake.

We fit all models with the *brms* package in R v. 4.0.4 (Bürkner, 2018; R Core Team, 2018). The predictive value of each model was determined with Bayesian stacking weights calculated by the *loo* package (Yao et al., 2018); conceptually, this is similar to AIC model weighting. A combined ensemble was created by pooling a weighted sample of each model's posterior distribution. We defined effect sizes as the standard deviation of a term's marginal effects at each posterior sample from a model where the term was included. Prior specification and other details are provided in the supplementary material section.

## 3 RESULTS

**FIGURE 2:**
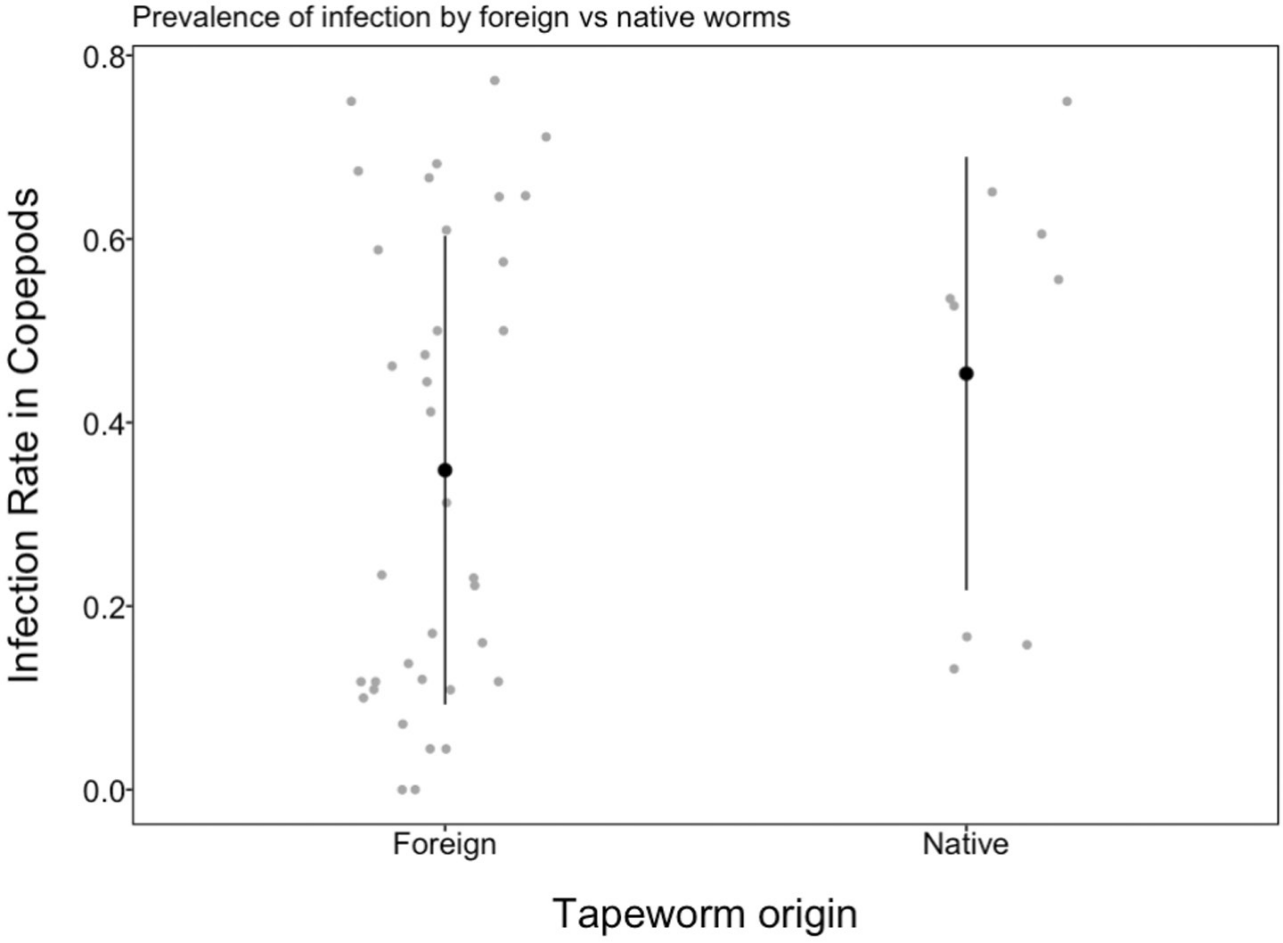
Overall, infection rates on copepods by local or native tapeworms (i.e. where the *S. solidus* tapeworms are from the same lakes as the copepods) are very similar to that of foreign tapeworms.

**FIGURE 3:**
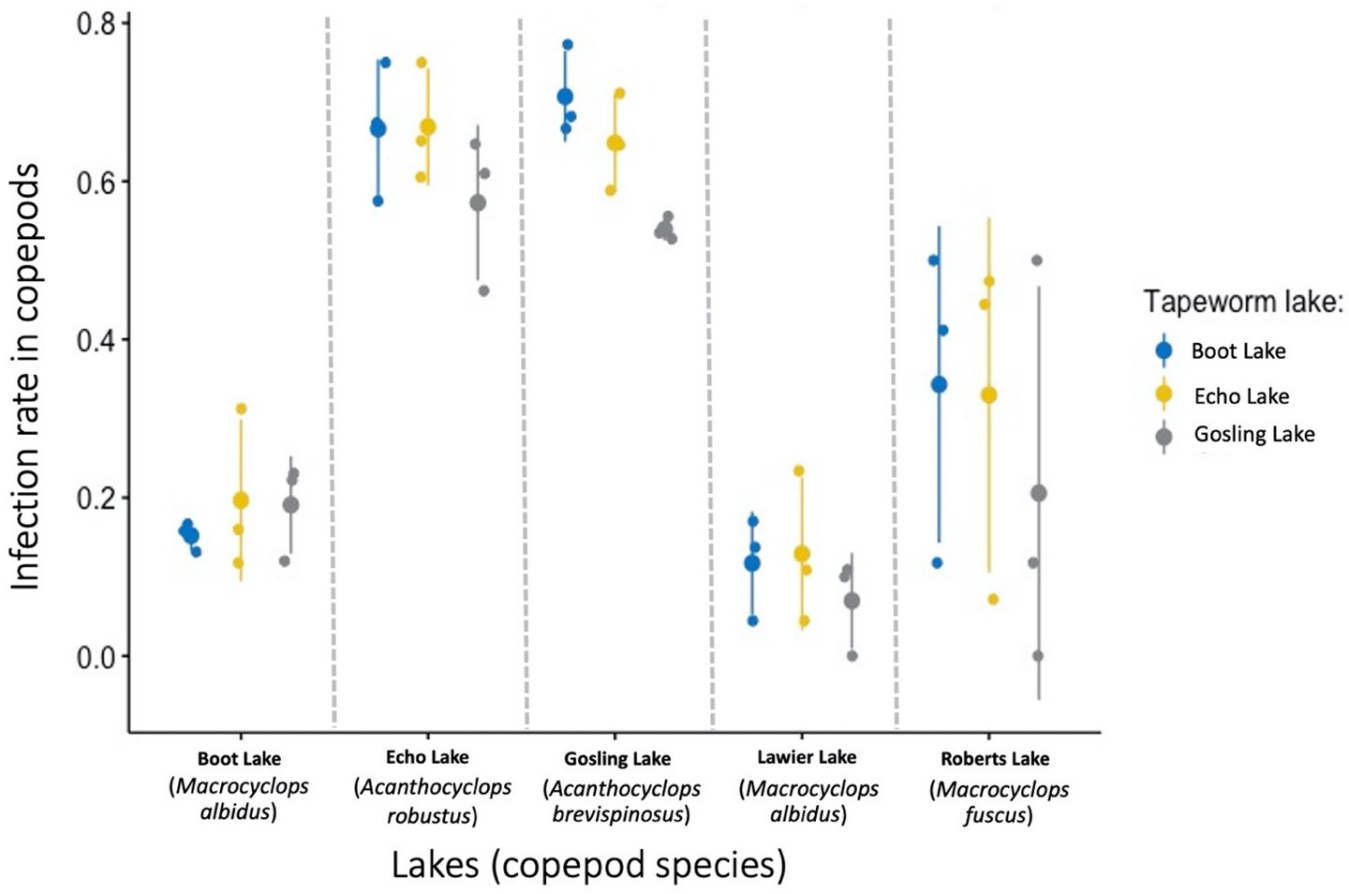
The infection rates in copepods were similar among all the tapeworm families or strains from the three lakes used. Copepods from Echo Lake and Gosling Lake (both Acanthocyclops) were 3–6 fold more susceptible to infection than the copepods from Boot, Echo, or Roberts Lakes (all *Macrocyclops* sp.). The scientific names of the copepods from each lake are in parenthesis under the lake names. The tapeworm is not found in Lawier and Roberts lakes (at least from stickleback fish surveys).

**FIGURE 4:**
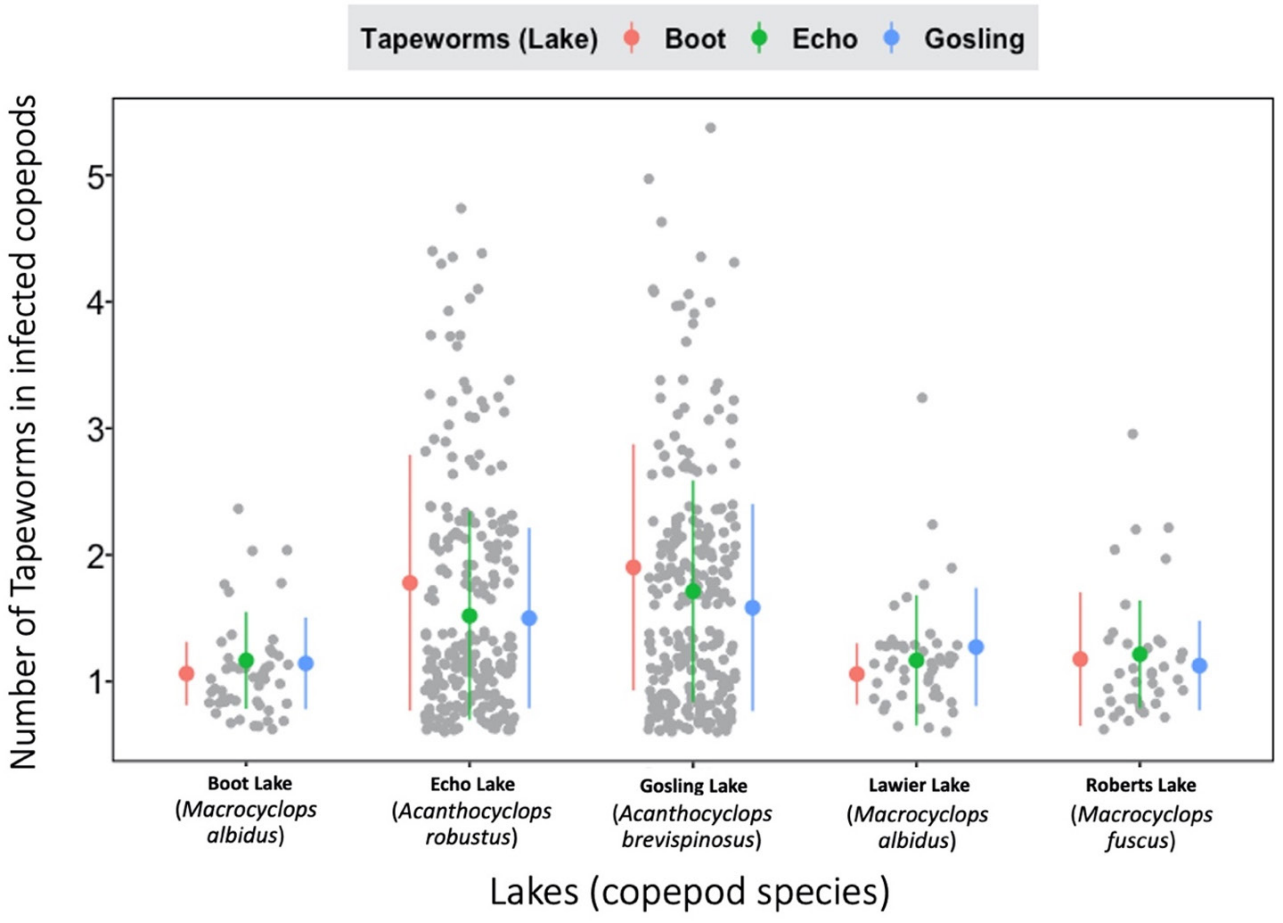
The infected Acanthocyclops copepods from Echo and Gosling Lakes also had slightly more parasites on average than the *Macrocyclops* copepods from the other three lakes. Again, the averages were very similar in the three tapeworm strains from the three lakes used. The scientific names of the copepods from each lake are in parenthesis under the lake names. The tapeworm is not found in Lawier and Roberts lakes (at least from stickleback fish surveys).

Our results indicate that *S. solidus* tapeworms from our study populations are not locally adapted to their native copepods (Figure 2), as infection rates similar when comparing sympatric copepod‐cestode pairs, versus allopatric copepod‐cestode pairs. However, *S. solidus* are moderately host specific: rates of infection and intensity (number of parasites inside infected hosts) were higher in *Acanthocyclops* than *Macrocyclops* copepods (Figures 3 and 4) regardless of which lake the parasites (or copepods) came from.

The ensemble mixed‐effect hurdle model contained 1.37 million posterior samples, with 4450 different models contributing at least one sample. No single model had a stacking weight higher than 0.3%; however, both copepod and tapeworm origins contributed to over 80% of both the intensity and infection rate model components (Table 1). For both model components, copepod type had the largest effect size of any term (intensity: 0.640 [0.421, 1.289]; infection rate: 0.255 [0.213, 0.364]; brackets signify 95% credible interval). Copepod effects can be decomposed into genus and lake of origin, with lake nested in genus; 61% of posterior samples with copepod genus terms also contained copepod lake. Copepod genus effect sizes were generally smaller when lake effects were also present (intensity effect sizes: 0.712 [0.518, 0.958] without lake, 0.342 [0.011, 2.714] with lake; infection rate: 0.338 [0.303, 0.375] without lake, 0.210 [0.031, 0.395] with lake; Figure S1).

**TABLE 1:** Inclusion frequencies and effect sizes of each term in the ensemble model for intensity (tapeworm count in infected copepods) and infection rate (prevalence). Effect sizes are provided as medians with 95% credible intervals and were calculated over the portion of the ensemble posterior where terms were present. Pooled terms indicate the combined effects of all copepod or tapeworm terms that were present in the model. Random effects are noted with (RE). Effect sizes for intensity and infection rate should not be compared, as they are in different units (counts and proportions, respectively).

Model component^a^	Term^b^	Ensemble frequency^c^	Effect size^d^	[95% CI]^e^
Intensity	Copepod (pooled)	0.849	0.640	[0.421, 1.289]
Intensity	Copepod Genus	0.849	0.621	[0.019, 2.201]
Intensity	Worm (pooled)	0.817	0.187	[0.027, 1.297]
Intensity	Worm Lake	0.817	0.182	[0.023, 1.374]
Intensity	Copepod lake	0.522	0.503	[0.190, 2.464]
Intensity	Plate (RE)	0.500	0.095	[0.004, 0.421]
Intensity	Native	0.473	0.126	[0.005, 0.798]
Intensity	Worm family (RE)	0.420	0.113	[0.006, 0.575]
Intensity	Genus × worm lake interaction	0.354	0.616	[0.052, 4.309]
Intensity	Copepod × worm lake interaction	0.208	0.721	[0.079, 7.482]
Infection rate	Copepod (pooled)	0.871	0.255	[0.213, 0.364]
Infection rate	Copepod genus	0.871	0.274	[0.043, 0.385]
Infection rate	Worm (pooled)	0.840	0.058	[0.020, 0.118]
Infection rate	Worm lake	0.840	0.060	[0.018, 0.128]
Infection rate	Copepod lake	0.593	0.143	[0.072, 0.242]
Infection rate	Plate (RE)	0.500	0.032	[0.002, 0.078]
Infection rate	Worm family (RE)	0.409	0.018	[0.001, 0.060]
Infection rate	Native	0.372	0.039	[0.002, 0.142]
Infection rate	Copepod × Worm lake interaction	0.235	0.095	[0.045, 0.164]
Infection rate	Genus × Worm lake interaction	0.218	0.053	[0.009, 0.175]

^a^ For Model Component; Infection Rate is the Prevalence of infection in copepods by the tapeworm. Intensity is the number of tapeworms inside infected copepods.

^b^ Factor indicates the model term; Copepod (pooled) and Worm (pooled) refer to the combination of whatever copepod/worm related terms were present in each model.

^c^ Frequency the proportion of the model ensemble that the term appears in.

^d^ Effect size of each model term accounting for the data.

^e^ CI: 95% credible interval of the effect size.

Copepod by tapeworm interactions (the typical test for local adaptation) had the lowest ensemble inclusion frequencies for both the intensity and infection rate model components (Table 1), and their effect sizes when present had wide, noisy posterior distributions. The ‘native’ effect (indicating copepods and tapeworms from the same lake) had higher inclusion frequency but consistently small effect sizes; we interpreted this as insufficient evidence for local adaptation. All of these effects had lower inclusion rates than the blocking effect of the 6‐well plates used for in the experiment.

We also ran mixed‐effect linear and GLM models in R (R Core Team) to supplement the analyses and results above. For these analyses, the best predictors for infection rate were the copepod and tapeworm source lakes, and the best predictor for intensity in infected copepods was copepod lake. These results did not differ considerably from the best Bayesian mixed‐effect hurdle models above, suggesting our results are robust to either choice of analytical method. For more details on the mix‐effect and GLM models and results, see Appendix S1.

As reported above, there was not enough evidence for local adaptation of the tapeworm to their copepod hosts. This can also be seen in Figure 2, where infection rates by the tapeworm on local (from the same lake) and foreign (from different lakes) copepods were very similar. However, there was evidence of host specificity as copepod genus was a strong predictor in infection rate and infection intensity in the crustacean. For example, copepods from Echo and Gosling lakes (both of the genus *Acanthocyclops*) were three to six times more susceptible to infection than the other copepod genus (*Marcocyclops*) from the three remaining lakes (Figure 3). This was true for all tapeworm strains used (Figure 3). Moreover, the infected copepods from Echo and Gosling lakes (again both of the genus *Acanthocyclops*) also had between 0.3‐ to 0.5‐fold more tapeworms than those (of the genus *Marcocyclops)* from the other three lakes (Figure 4). This accounts for the relatively high effect sizes of the copepod genus factor in the Bayesian analysis.

## 4 DISCUSSION

We tested for local adaptation and host specificity of the tapeworm *S. solidus* from three lakes in Vancouver Island to copepods from the same lakes, plus two more lakes where the tapeworm is absent (Weber, Steinel, et al., 2017). Researchers argue that parasites with complex life cycles should be more host‐specific (Noble et al., 1989; Poulin, 2007), and that parasites with higher dispersal rates should locally adapt to their hosts (Barber & Scharsack, 2009). Thus, the *S. solidus* tapeworm, being a parasite with a complex life cycle and having higher dispersal rates than their intermediate hosts (Dubinina, 1980), should show local adaptation and host specificity to its copepod hosts in a similar fashion to the tapeworm's second intermediate host (i.e. threespine sticklebacks [Hafer, 2018; Weber, Kalbe, et al., 2017; Kalbe et al., 2016]).

However, our results indicate that there was no evidence of differences between infection rates by local and foreign tapeworms on the copepods (Figure 2). The same is true for infection intensity, providing further confirmation of our conclusions. Our experiment also shows that copepods from Echo and Gosling Lakes (genus *Acanthocyclops*) were more susceptible to *S. solidus* tapeworm infection than the ones from the other three lakes (genera *Macrocyclops*) (Figure 3). Again, infection intensity corroborates our results from infection rates: *Acanthocyclops* copepods also had slightly more tapeworms when infected (Figure 4). These infection and intensity rates were very similar among the different tapeworm strains from the three lakes used (Figures 3 and 4). Thus, at least for this parasite–host system, we did not observe local adaptation by the tapeworm to the copepods.

Instead, the success of the tapeworm within a given lake depended mostly on whether a copepod genus (*Acanthocyclops*) was present. The higher susceptibility of Echo and Gosling Lakes' copepods (of the genus *Acanthocyclops*) to the tapeworm explains why copepod and tapeworm lake variables in our models best fit the data. This variation in zooplankton community structure, between lakes, means that tapeworm eggs deposited into lakes with *Macrocyclops* spp copepods will be less likely to transmit to their second intermediate host. Geographic variation in zooplankton community composition among lakes therefore can play a large role in generating among‐lake variation in infection for stickleback and ultimately piscivorous birds like loons.

To emphasize more the lack of local adaptation in our experiments, Boot and Lawier Lakes had the same species of copepods (*Macrocyclops albidus*), but both lakes' copepods had very similar infections rates by the three strains of tapeworms used (Figure 4). Specifically, Boot Lake tapeworms are no more (or less) effective at infecting Boot Lake *M. albidus* than they are at infected Lawier Lake *M. albidus* (a home‐versus‐away criterion for local (mal)adaptation). Nor are the Boot Lake tapeworms any better (or worse) at infecting their native Boot Lake copepods, relative to tapeworms from two other lakes (a native versus immigrant criterion for local (mal)adaptation). Thus, for both lakes, the infection rate by local tapeworms was not significantly different to that of foreign tapeworms.

Local adaptation aside, our experiments show the tapeworm is clearly capable of infecting multiple copepod genera, but it is most efficient at infecting a particular genus. The copepods with the highest infection rates (those from Echo and Gosling lakes) were from the same genus (i.e. *Acanthocyclops*). This was true regardless of whether the tapeworms were taken from a lake dominated by *Acanthocyclops*, or not. Currently, *M. albidus* copepods are most often used for experimental lab infections in sticklebacks (Barber, 2013; Benesh, 2010; Smyth, 1990; Weber, Kalbe, et al., 2017; Weber, Steinel, et al., 2017; Wedekind, 1997); perhaps, future work should employ *Acanthocyclop* species instead to maximize resources and time for better results. Weber, Kalbe, et al. (2017) showed that tapeworm infection prevalences (and intensities) vary greatly between stickleback populations, even between adjacent lakes. To explain this geographic heterogeneity, Weber et al suggested researchers investigate geographic variation in stickleback diet (copepod consumption rates), infection rates within these copepods, and host immunity once a parasite is ingested. Data from a few lakes confirms that both fish diet and immune genetics plays a role (Weber, Steinel, et al., 2017), but the role of variation in copepod infection rates has been overlooked. Our results (using some of the same lakes studied by Weber, Kalbe, et al., 2017; Weber, Steinel, et al., 2017), confirms that copepods differ in their susceptibility to *S. solidus* infections. In particular, lakes with *Acanthocyclops* copepods might become hotspots for stickleback infections, because those copepods are more effective hosts for the tapeworm. While we would need to sample more lakes to solidify this conclusion, it is noteworthy that the two lakes with *Acanthocyclops* (Gosling and Echo Lakes) both have especially high stickleback infection rates compared to many other lakes (Figure 1 in Weber, Steinel, et al., 2017; Weber, Kalbe, et al., 2017). Our results reveal a previously under‐appreciated source of geographic variation in the epidemiology of *S. solidus* parasites. More generally, this result illustrates how understanding the epidemiology of multi‐host parasites requires experimental studies of infection dynamics across all available hosts.

## AUTHOR CONTRIBUTIONS

**Kum Chuan Shim:** Conceptualization (lead); data curation (lead); formal analysis (equal); funding acquisition (lead); investigation (lead); methodology (lead); visualization (lead); writing – original draft (lead); writing – review and editing (lead). **Christopher R. Peterson:** Formal analysis (equal); visualization (equal); writing – original draft (supporting); writing – review and editing (supporting). **Daniel I. Bolnick:** Conceptualization (supporting); formal analysis (supporting); writing – review and editing (supporting).

## FUNDING INFORMATION

This work was funded by the Graduate Program in Ecology and Evolutionary Biology at the University of Texas at Austin, and an NSERC Graduate Research Fellowship to WS.

## Supporting information


Appendix S1

## Data Availability

All data and code needed to reproduce the results of this study are archived on Dryad at https://doi.org/10.5061/dryad.w9ghx3ftz.
