# Supplementary material for: Local adaptation and host specificity to copepod intermediate hosts by the tapeworm Schistocephalus solidus
Source: Ecol Evol. 2023 Jun 6;13(6):e10155. doi: 10.1002/ece3.10155 (PMC10242650; doi:10.1002/ece3.10155)
Supplement: Supplementary file 1 — Appendix S1 [file ECE3-13-e10155-s001.docx]

**Supplementary material**

Supplementary table 1: copepod collection dates and locality:

| **Lake** | **Longitude** | **Latitude** | **Collection date** |
| --- | --- | --- | --- |
| Boot Lake | 50.05503 | -125.526 | 9/16/17, 6/24/18 |
| Echo Lake | 49.98765 | -125.411 | 9/15/17, 6/24/18 |
| Lawier Lake | 50.083144 | -125.515052 | 9/15/17, 6/24/18 |
| Roberts Lake | 50.216390 | -125.544687 | 9/15/17, 6/24/18 |
| Gosling Lake | 50.04592 | -125.501 | 9/16/17, 6/24/18 |

Supplementary table 2: Tapeworm families used for the experiments and number of exposures (i.e. wells in a 6-well plate) for each lake’s copepods (reminder: each well had 10 copepods exposed to 20 tapeworms):

| **Tapeworm families^1^** | **Gosling Lake copepods** | **Roberts Lake copepods** | **Lawier Lake copepods** | **Echo Lake copepods** | **Boot Lake copepods** |
| --- | --- | --- | --- | --- | --- |
| Control | 6 | 6 | 6 | 8 | 7 |
| Boot 11Bx1A (6/17/18) | 6 | 6 | 6 | 5 | 6 |
| Boot 2Ax2C (6/17/18) | 6 | 6 | 5 | 4 | 6 |
| Boot bulk  (7/3/18) | 6 | 6 | 5 | 5 | 6 |
| Echo bulk  (6/15/18) | 6 | 6 | 6 | 5 | 6 |
| Echo 3Ax1A  (6/10/18) | 6 | 6 | 6 | 5 | 6 |
| Echo 27Ax31A  (6/17/18) | 5 | 6 | 5 | 6 | 6 |
| Gosling 7Ax1A  (6/10/18) | 6 | 6 | 6 | 5 | 6 |
| Gosling 10Ax12A  (6/17/18) | 6 | 7 | 5 | 5 | 5 |
| Gosling 2  (9/22/18) | 6 | 6 | 6 | 5 | 6 |

^1^ Tapeworm families used in the experiment; 3 families per lake; dates in the parentheses below each family indicate the time the tapeworm eggs were harvested in the lab for the experiment. Control indicates no tapeworms were used to exposed the copepods (i.e. negative control); this is to evaluate the survivorship of the copepods in the wells during the experiment.

**
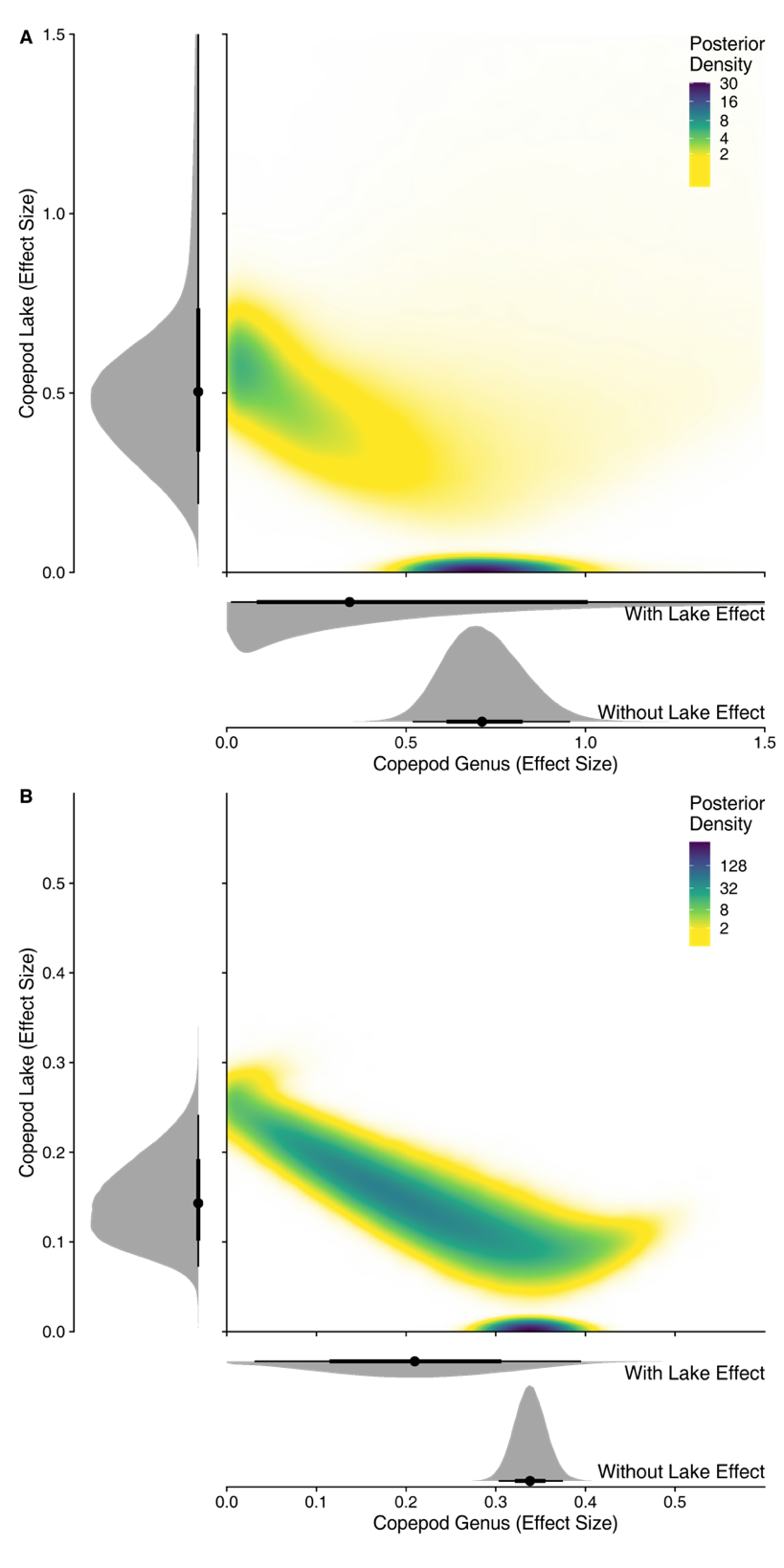
**

Supplementary figure **1**: Distribution of effect sizes for Copepod Genus and Copepod Lake for the infection intensity (**A**) and rate (**B**) model components. Both panels include the marginal effects of copepod lake (left), the marginal effects of copepod genus conditioned on whether lake was included in the model (bottom), and their bivariate distributions (upper right). For both terms, the genus effect increases when the lake effect declines or is absent; this is particularly notable for the intensity component.


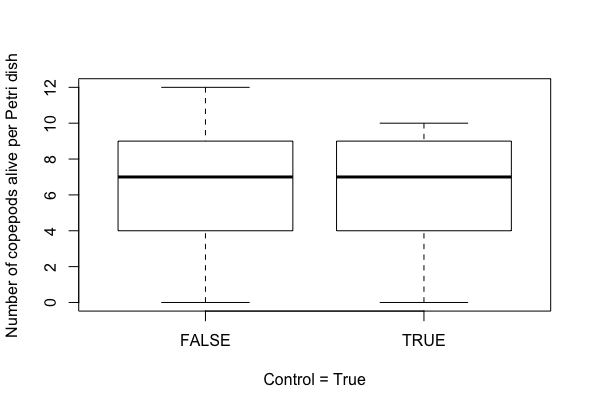


Supplementary figure **2:** The number of copepods alive after termination of experiment did not differ significantly between those exposed to the tapeworm and those that were not (i.e., control) [P value = 0.996, see more details of analysis in Supplementary mix-effect linear and GLM model analyses below]

**Supplementary information on the Bayesian Analysis**

**Priors and iterations used in the mixed-effect hurdle analysis:**

Each model was run for 4 chains with 1000 warmup and 1000 sampling iterations each. We checked model convergence by verifying N_eff > 1000, R-hat > 1.01, and all Hamiltonian Monte-Carlo diagnostics were acceptable.

Our prior distributions were Normal(mean = 0, sd = 6) priors for the intercepts of both model components. For incidence, we used Normal(0,1) priors for fixed effect coefficients and half-t(df = 7, mean = 0, scale = 1) priors for the standard deviation of the random effects. For the prevalence model, our priors were Normal(0, 1.5) for fixed effects and half-t(7, 0, 1.5) for the random effects. These priors were selected because they were flexible enough to allow for large effects but conservative enough to avoid spurious results.

Note: The complete R script for the mixed-effect hurdle analyses is in Christopher Peterson’s GitHub (**https://github.com/Christopher-Peterson/copepod_worm_adapt**).

**Supplementary mix-effect linear and GLM model** **analyses:**

I) Analyzing if there was any difference on survival rate between copepods exposed to tapeworms and the not-exposed ones (i.e. control):

> model15 = glm(cop.alive ~ is_control, data = control_df)

> anova(model15, test = "LRT")

Analysis of Deviance Table

Model: Poisson, link: log

Response: cop.alive

Terms added sequentially (first to last)

Df Deviance Resid. Df Resid. Dev Pr(>Chi)

NULL 291 2409.6

is_control 1 0.0001767 290 2409.6 **0.9963**

**Summary: not significant difference on number of copepods alive after experiment from control vs. exposed copepods (i.e. exposed to tapeworms)**

> model14 = glm(cop.death.numb ~ is_control, data = control_df, family = poisson())

> anova(model14, test = "LRT")

Analysis of Deviance Table

Model: poisson, link: log

Response: cop.death.numb

Terms added sequentially (first to last)

Df Deviance Resid. Df Resid. Dev Pr(>Chi)

NULL 291 684.6

is_control 1 0.00056907 290 684.6 **0.981**

**Summary: not significant difference on number of copepod deaths during experiment from control vs. exposed copepods (i.e. exposed to tapeworms)**

**II). GLM and GLMM analyses companion to the Bayesian analysis:**

Using lme4 version 1.1-13 package for R

> summary(model1)

Generalized linear mixed model fit by maximum likelihood (Laplace Approximation) [

glmerMod]

Family: binomial ( logit )

Model 1 : infected.yes.no ~ cop.lake * worm.lake + (1 | plate)

Data: copepods

AIC BIC logLik deviance df.resid

1754.9 1841.1 -861.4 1722.9 1606

Scaled residuals:

Min 1Q Median 3Q Max

-1.6429 -0.4962 -0.3703 0.7090 3.7777

Random effects:

Groups Name Variance Std.Dev.

plate (Intercept) 0.02598 0.1612

Number of obs: 1622, groups: plate, 51

Fixed effects:

Estimate Std. Error z value Pr(>|z|)

(Intercept) -1.7593 0.2789 -6.308 2.83e-10 ***

cop.lakeech 2.4600 0.3361 7.318 2.51e-13 ***

cop.lakegos 2.6596 0.3460 7.686 1.52e-14 ***

cop.lakelau -0.1886 0.3737 -0.505 0.61378

cop.lakerob 1.0933 0.4011 2.726 0.00642 **

worm.lakeech 0.3491 0.3839 0.909 0.36318

worm.lakegos 0.3241 0.4025 0.805 0.42065

cop.lakeech:worm.lakeech -0.3245 0.4721 -0.687 0.49182

cop.lakegos:worm.lakeech -0.6611 0.4740 -1.395 0.16310

cop.lakelau:worm.lakeech -0.3105 0.5246 -0.592 0.55388

cop.lakerob:worm.lakeech -0.3525 0.5873 -0.600 0.54837

cop.lakeech:worm.lakegos -0.7522 0.4870 -1.545 0.12246

cop.lakegos:worm.lakegos -1.0517 0.4811 -2.186 0.02881 *

cop.lakelau:worm.lakegos -0.9553 0.5728 -1.668 0.09537 .

cop.lakerob:worm.lakegos -1.1049 0.6368 -1.735 0.08271 .

---

Signif. codes: 0 ‘***’ 0.001 ‘**’ 0.01 ‘*’ 0.05 ‘.’ 0.1 ‘ ’ 1

Correlation matrix not shown by default, as p = 15 > 12.

Use print(x, correlation=TRUE) or

vcov(x) if you need it

convergence code: 0

Model failed to converge with max|grad| = 0.00320527 (tol = 0.001, component 1)

> anova(model1,test="LRT")

Analysis of Variance Table

Df Sum Sq Mean Sq F value

cop.lake 4 326.57 81.643 81.6425

worm.lake 2 12.22 6.110 6.1102

cop.lake:worm.lake 8 6.20 0.775 0.7746

**2) Doing another model without the interaction of copepod lakes and worm lakes:**

> model2 <- glmer(infected.yes.no ~ cop.lake + worm.lake + (1|plate),

+ data=copepods, family="binomial") #model 2 does not have an interaction

> #comparing models 1 and 2:

> anova(model1, model2)

Data: copepods

Models:

model2: infected.yes.no ~ cop.lake + worm.lake + (1 | plate)

model1: infected.yes.no ~ cop.lake * worm.lake + (1 | plate)

Df AIC BIC logLik deviance Chisq Chi Df Pr(>Chisq)

model2 8 1744.9 1788.0 -864.46 1728.9

model1 16 1754.9 1841.1 -861.43 1722.9 6.0436 8 0.6423

model2 without the interaction is slightly better! Though not significantly

**3) model 6, testing for GLM without taking into account the random variable (no random effect)**

> model6 <- glm(infected.yes.no ~ cop.lake + worm.lake + cop.lake*worm.lake, + data = copepods,family="binomial")

> summary(model6)

Call:

glm(formula = infected.yes.no ~ cop.lake + worm.lake + cop.lake *

worm.lake, family = "binomial", data = copepods)

Deviance Residuals:

Min 1Q Median 3Q Max

-1.5645 -0.6639 -0.5187 0.8962 2. 2917

Coefficients:

Estimate Std. Error z value Pr(>|z|)

(Intercept) -1.7272 0.2713 -6.366 1.94e-10 ***

cop.lakeech 2.4319 0.3292 7.388 1.49e-13 ***

cop.lakegos 2.6027 0.3302 7.882 3.21e-15 ***

cop.lakelau -0.2107 0.3704 -0.569 0.56938

cop.lakerob 1.0912 0.3982 2.740 0.00614 **

worm.lakeech 0.3271 0.3780 0.865 0.38677

worm.lakegos 0.2921 0.3952 0.739 0.45973

cop.lakeech:worm.lakeech -0.3263 0.4636 -0.704 0.48161

cop.lakegos:worm.lakeech -0.6018 0.4568 -1.318 0.18765

cop.lakelau:worm.lakeech -0.2863 0.5199 -0.551 0.58185

cop.lakerob:worm.lakeech -0.3843 0.5788 -0.664 0.50670

cop.lakeech:worm.lakegos -0.7143 0.4761 -1.500 0.13356

cop.lakegos:worm.lakegos -1.0181 0.4707 -2.163 0.03056 *

cop.lakelau:worm.lakegos -0.9051 0.5637 -1.606 0.10834

cop.lakerob:worm.lakegos -1.0732 0.6296 -1.705 0.08828 .

---

Signif. codes: 0 ‘***’ 0.001 ‘**’ 0.01 ‘*’ 0.05 ‘.’ 0.1 ‘ ’ 1

(Dispersion parameter for binomial family taken to be 1)

Null deviance: 2167.1 on 1621 degrees of freedom

Residual deviance: 1723.3 on 1607 degrees of freedom

AIC: 1753.3

Number of Fisher Scoring iterations: 5

> anova(model6,test="LRT") #"LRT" is to get P-values here to check quickly for significance for the variables in the model

Analysis of Deviance Table

Model: binomial, link: logit

Response: infected.yes.no

Terms added sequentially (first to last)

Df Deviance Resid. Df Resid. Dev Pr(>Chi)

NULL 1621 2167.1

cop.lake 4 424.73 1617 1742.4 < 2.2e-16 ***

worm.lake 2 13.35 1615 1729.0 0.001265 **

cop.lake:worm.lake 8 5.71 1607 1723.3 0.6 79743

---

Signif. codes: 0 ‘***’ 0.001 ‘**’ 0.01 ‘*’ 0.05 ‘.’ 0.1 ‘ ’ 1

**Note: From the results above, there’re very significant effects of copepod lake and worm lake, but no interaction between them.**

**4) testing for effect of plates: (summary answer after running the model below: plate does not matter)**

> model7 <- glm(infected.yes.no ~ cop.lake + worm.lake + cop.lake*worm.lake + plate,

+ data=copepods,family="binomial")

> anova(model6, model7) #does not give AIC comparisons, so this kind of useless

Analysis of Deviance Table

Model 1: infected.yes.no ~ cop.lake + worm.lake + cop.lake * worm.lake

Model 2: infected.yes.no ~ cop.lake + worm.lake + cop.lake * worm.lake +

plate

Resid. Df Resid. Dev Df Deviance

1 1607 1723.3

2 1557 1658.6 50 64.754

> summary(model7)

Call:

glm(formula = infected.yes.no ~ cop.lake + worm.lake + cop.lake *

worm.lake + plate, family = "binomial", data = copepods)

Deviance Residuals:

Min 1Q Median 3Q Max

-2.1391 -0.7205 -0.4181 0.8657 2.7258

Coefficients:

Estimate Std. Error z value Pr(>|z|)

(Intercept) -3.400215 1.128677 -3.013 0.00259 **

cop.lakeech 2.648247 0.376858 7.027 2.11e-12 ***

cop.lakegos 3.271272 0.396900 8.242 < 2e-16 ***

cop.lakelau 0.004914 0.393591 0.012 0.99004

cop.lakerob 1.103968 0.423390 2.607 0.00912 **

worm.lakeech 0.547379 0.427087 1.282 0.19996

worm.lakegos 0.650357 0.444572 1.463 0.14350

platep1 1.423063 1.203531 1.182 0.23704

platep10 1.183440 1.187952 0.996 0.31915

platep11 -0.330903 1.234766 -0.268 0.78871

platep12 1.690147 1.195298 1.414 0.15736

platep13 1.281547 1.180913 1.085 0.27783

platep14 2.257173 1.174100 1.922 0.05455 .

platep15 1.166814 1.219485 0.957 0.33866

platep16 0.387203 1.170179 0.331 0.74073

platep17 1.225190 1.179514 1.039 0.29893

platep18 2.003874 1.230927 1.628 0.10354

platep19 0.919460 1.176703 0.781 0.43457

platep2 1.260738 1.188751 1.061 0.28889

platep20 0.943129 1.180947 0.799 0.42451

platep21 2.117863 1.184146 1.789 0.07369 .

platep22 0.870342 1.179558 0.738 0.46060

platep23 2.369486 1.170017 2.025 0.04285 *

platep24 1.636683 1.212048 1.350 0.17691

platep25 1.688014 1.198811 1.408 0.15911

platep26 1.536905 1.196642 1.284 0.19902

platep27 1.158677 1.194184 0.970 0.33191

platep28 1.765654 1.176215 1.501 0.13332

platep29 1.696665 1.231557 1.378 0.16831

platep3 1.741903 1.187559 1.467 0.14243

platep30 0.459434 1.208949 0.380 0.70393

platep31 0.996894 1.182753 0.843 0.39931

platep32 1.586515 1.188720 1.335 0.18199

platep33 1.394902 1.237889 1.127 0.25981

platep34 1.438833 1.154879 1.246 0.21281

platep35 0.365781 1.220012 0.300 0.76432

platep36 1.320014 1.189100 1.110 0.26696

platep37 1.164217 1.172606 0.993 0.32079

platep38 1.558819 1.183279 1.317 0.18771

platep39 1.482254 1.204915 1.230 0.21863

platep4 2.309692 1.206378 1.915 0.05555 .

platep40 1.950804 1.181498 1.651 0.09871 .

platep41 1.305689 1.223933 1.067 0.28606

platep42 Estimate Std. Error z value Pr(>|z|)

(Intercept) -3.3798150 1.1267879 -3.000 0.00270 **

cop.lakeech 2.6629963 0.3773947 7.056 1.71e-12 ***

cop.lakegos 3.2849437 0.3973952 8.266 < 2e-16 ***

cop.lakelau -0.0526042 0.3916659 -0.134 0.89316

cop.lakerob 1.1102455 0.4235788 2.621 0.00876 **

worm.lakeech 0.5649020 0.4274367 1.322 0.18630

worm.lakegos 0.6713093 0.4449921 1.509 0.13140

platep1 1.4001004 1.2023205 1.164 0.24422

platep10 1.1421783 1.1859406 0.963 0.33550

platep11 -0.3649309 1.2333211 -0.296 0.76731

platep12 1.6596767 1.1936453 1.390 0.16440

platep13 1.2550194 1.1795068 1.064 0.28732

platep14 2.2249946 1.1725085 1.898 0.05774 .

platep15 1.1187589 1.2173953 0.919 0.35811

platep16 0.3611196 1.1689989 0.309 0.75739

platep17 1.1901914 1.1777752 1.011 0.31224

platep18 1.9470174 1.2285226 1.585 0.11300

platep19 0.9021327 1.1759706 0.767 0.44300

platep2 1.2408435 1.1877500 1.045 0.29616

platep20 0.9219882 1.1799472 0.781 0.43458

platep21 2.1044216 1.1832326 1.779 0.07532 .

platep22 0.8400550 1.1781515 0.713 0.47583

platep23 2.3557240 1.1686849 2.016 0.04383 *

platep24 1.5905510 1.2096493 1.315 0.18855

platep25 1.6344116 1.1964111 1.366 0.17191

platep26 1.4913152 1.1944169 1.249 0.21182

platep27 1.1385838 1.1933126 0.954 0.34001

platep28 1.7516352 1.1750112 1.491 0.13603

platep29 1.6434851 1.2292294 1.337 0.18122

platep3 1.6953214 1.1854924 1.430 0.15270

platep30 0.4280207 1.2074214 0.354 0.72297

platep31 0.9763900 1.1818188 0.826 0.40870

platep32 1.5603954 1.1872418 1.314 0.18874

platep33 1.3499887 1.2353393 1.093 0.27448

platep34 1.4182930 1.1539588 1.229 0.21905

platep35 0.3351304 1.2185258 0.275 0.78329

platep36 1.2740963 1.1869288 1.073 0.28307

platep37 1.1311015 1.1709521 0.966 0.33406

platep38 1.5187579 1.1813184 1.286 0.19857

platep39 1.4487093 1.2032578 1.204 0.22859

platep4 2.2814105 1.2049449 1.893 0.05831 .

platep40 1.9408945 1.1805980 1.644 0.10018

platep41 1.2763272 1.2225738 1.044 0.29650

platep42 1.3990490 1.1997577 1.166 0.24357

platep43 0.7191183 1.1960971 0.601 0.54769

platep44 1.1219854 1.1798769 0.951 0.34164

platep45 1.2439503 1.1811677 1.053 0.29227

platep46 1.0435061 1.1943221 0.874 0.38227

platep47 0.8521671 1.2247366 0.696 0.48656

platep48 1.0256932 1.2030773 0.853 0.39390

platep49 1.5725579 1.1901917 1.321 0.18641

platep5 2.1948244 1.1726097 1.872 0.06124 .

platep50 2.1848215 1.1983475 1.823 0.06827 .

platep6 1.1904317 1.1870980 1.003 0.31595

platep7 1.8411879 1.2012220 1.533 0.12533

platep8 1.1430506 1.2024364 0.951 0.34180

platep9 1.3928382 1.2060656 1.155 0.24815

cop.lakeech:worm.lakeech -0.1959257 0.5569642 -0.352 0.72501

cop.lakegos:worm.lakeech -1.4267929 0.5652912 -2.524 0.01160 *

cop.lakelau:worm.lakeech -0.4480476 0.5534265 -0.810 0.41818

cop.lakerob:worm.lakeech 0.0002931 0.6431409 0.000 0.99964

cop.lakeech:worm.lakegos -1.1915784 0.5580610 -2.135 0.03274 *

cop.lakegos:worm.lakegos -1.5504693 0.5540004 -2.799 0.00513 **

cop.lakelau:worm.lakegos -1.3602538 0.6179272 -2.201 0.02771 *

cop.lakerob:worm.lakegos -1.4027838 0.6821333 -2.056 0.03974 *

---

Signif. codes: 0 ‘***’ 0.001 ‘**’ 0.01 ‘*’ 0.05 ‘.’ 0.1 ‘ ’ 1

(Dispersion parameter for binomial family taken to be 1)

Null deviance: 2167.1 on 1621 degrees of freedom

Residual deviance: 1658.6 on 1557 degrees of freedom

AIC: 1788.6

Number of Fisher Scoring iterations: 5

> anova(model7, test = "LRT")

Analysis of Deviance Table

Model: binomial, link: logit

Response: infected.yes.no

Terms added sequentially (first to last)

Df Deviance Resid. Df Resid. Dev Pr(>Chi)

NULL 1621 2167.1

cop.lake 4 424.73 1617 1742.4 < 2.2e-16 ***

worm.lake 2 13.35 1615 1729.0 0.00 1265 **

plate 50 54.14 1565 1674.9 0.319232

cop.lake:worm.lake 8 16.52 1557 1658.6 0.038042 *

---

Signif. codes: 0 ‘***’ 0.001 ‘**’ 0.01 ‘*’ 0.05 ‘.’ 0.1 ‘ ’ 1

Note: no significance in the plates, so plate does not have an effect in the infection outcome. But weird that there’s a slight significant interaction on copepod and worm lake interactions

**5) testing for local adaptation**

> model8 <- glm(infected.yes.no ~ cop.lake + worm.lake + native, + data=copepods, family="binomial")

> summary(model8)

Call:

glm(formula = infected.yes.no ~ cop.lake + worm.lake + native,

family = "binomial", data = copepods)

Deviance Residuals:

Min 1Q Median 3Q Max

-1.5174 -0.6755 -0.5094 0.8875 2.2339

Coefficients:

Estimate Std. Error z value Pr(>|z|)

(Intercept) -1.34434 0.18042 -7.451 9.24e-14 ***

cop.lakeech 2.11536 0.19279 10.972 < 2e-16 ***

cop.lakegos 2.08992 0.18921 11.045 < 2e-16 ***

cop.lakelau -0.61487 0.22859 -2.690 0.00715 **

cop.lakerob 0.61262 0.25303 2.421 0.01547 *

worm.lakeech -0.01723 0.14308 -0.120 0.90415

worm.lakegos -0.44988 0.14882 -3.023 0.00250 **

nativeTRUE -0.15688 0.14466 -1.084 0.27817

---

Signif. codes: 0 ‘***’ 0.001 ‘**’ 0.01 ‘*’ 0.05 ‘.’ 0.1 ‘ ’ 1

(Dispersion parameter for binomial family taken to be 1)

Null deviance: 2167.1 on 1621 degrees of freedom

Residual deviance: 1727.8 on 1614 degrees of freedom

AIC: 1743.8

Number of Fisher Scoring iterations: 4

Df Deviance Resid. Df Resid. Dev Pr(>Chi)

NULL 1621 2167.1

cop.lake 4 424.73 1617 1742.4 < 2.2e-16 ***

worm.lake 2 13.35 1615 1729.0 0.001073 **

native 1 1.18 1614 1727.8 **0.278190**

---

Signif. codes: 0 ‘***’ 0.001 ‘**’ 0.01 ‘*’ 0.05 ‘.’ 0.1 ‘ ’ 1

**Summary: no local adaptation**

**6) testing for effect of worm family used: (summary answer after running the model below: tapeworm family does not matter)**

> model9 <- glm(infected.yes.no ~ cop.lake + worm.lake + cop.lake*worm.lake + worm.fam,

+ data=copepods,family="binomial")

> summary(model9)

Call:

glm(formula = infected.yes.no ~ cop.lake + worm.lake + cop.lake *

worm.lake + worm.fam, family = "binomial", data = copepods)

Deviance Residuals:

Min 1Q Median 3Q Max

-1.5972 -0.6913 -0.4666 0.8940 2.3145

Coefficients: (2 not defined because of singularities)

Estimate Std. Error z value Pr(>|z|)

(Intercept) -1.66090 0.30207 -5.498 3.83e-08 ***

cop.lakeech 2.43654 0.32947 7.395 1.41e-13 ***

cop.lakegos 2.60899 0.33064 7.891 3.00e-15 ***

cop.lakelau -0.20226 0.37076 -0.546 0.58540

cop.lakerob 1.09678 0.39853 2.752 0.00592 **

worm.lakeech 0.42579 0.42204 1.009 0.31303

worm.lakegos 0.13006 0.44256 0.294 0.76885

worm.famboo2ax2c -0.15686 0.24765 -0.633 0.52648

worm.famboobulk -0.06496 0.24469 -0.265 0.79066

worm.famech27ax31a -0.41496 0.25145 -1.650 0.09888 .

worm.famech3ax1a -0.07461 0.24443 -0.305 0.76019

worm.famechbulk NA NA NA NA

worm.famg10ax12a 0.04772 0.25413 0.188 0.85104

worm.famg2 0.23203 0.25941 0.894 0.37107

worm.famg7ax1a NA NA NA NA

cop.lakeech:worm.lakeech -0.33392 0.46483 -0.718 0.47253

cop.lakegos:worm.lakeech -0.60388 0.45816 -1.318 0.18749

cop.lakelau:worm.lakeech -0.31071 0.52098 -0.596 0.55091

cop.lakerob:worm.lakeech -0.43645 0.58100 -0.751 0.45253

cop.lakeech:worm.lakegos -0.70794 0.47683 -1.485 0.13763

cop.lakegos:worm.lakegos -1.00586 0.47187 -2.132 0.03304 *

cop.lakelau:worm.lakegos -0.92188 0.56421 -1.634 0.10227

cop.lakerob:worm.lakegos -1.06786 0.63030 -1.694 0.09023 .

---

Signif. codes: 0 ‘***’ 0.001 ‘**’ 0.01 ‘*’ 0.05 ‘.’ 0.1 ‘ ’ 1

(Dispersion parameter for binomial family taken to be 1)

Null deviance: 2167.1 on 1621 degrees of freedom

Residual deviance: 1727.2 on 1599 degrees of freedom

AIC: 1773.2

Number of Fisher Scoring iterations: 13

> anova(model9, test = "LRT")

Analysis of Deviance Table

Model: binomial, link: logit

Response: infected.yes.no

Terms added sequentially (first to last)

Df Deviance Resid. Df Resid. Dev Pr(>Chi)

NULL 1621 2167.1

cop.lake 4 424.73 1617 1742.4 < 2.2e-16 ***

worm.lake 2 13.35 1615 1729.0 0.001265 **

worm.fam 8 0.74 1607 1728.3 0.999425

cop.lake:worm.lake 8 1.07 1599 1727.2 0.997795

---

Signif. codes: 0 ‘***’ 0.001 ‘**’ 0.01 ‘*’ 0.05 ‘.’ 0.1 ‘ ’ 1

**Summary: worm family does not matter!**

**Comparing all models on testing the prevalence of infection:**

> AIC(model1, model2, model3,model4, model5, model6, model7, model8, model9)

Note: according to lab-mate Christopher Peterson, it is fine to do AIC comparisons between GLM and GLMM models (need to ask him for the reference).

Below are the models sorted from best to worst: (the first number after each model name is the degrees of freedom followed by the Akaike Information Criterion (AIC) value:

**df AIC**

**1) model8** 8 1743.843

model8 <- glm(infected.yes.no ~ cop.lake + worm.lake + native, data = copepods, family = "binomial") #testing for local adaptation

**2) model2** 8 1744.910

model2 <- glmer(infected.yes.no ~ cop.lake + worm.lake + (1|plate),

data=copepods,family="binomial") #GLMM not testing for interaction

**3) model6** 15 1753.310

model6 <- glm(infected.yes.no ~ cop.lake + worm.lake + cop.lake*worm.lake, data = copepods,family="binomial") #GLM testing for interactions between cop.lake and worm.lake

**4) model3** 6 1753.846

model3 <- glmer(infected.yes.no ~ cop.lake + (1|plate), data = copepods, family = "binomial") #testing for copepod lake only

**5) model1** 16 1754.866

model1 <- glmer(infected.yes.no ~ cop.lake*worm.lake + (1|plate), data = copepods, family = "binomial") #testing for effects of cop.lake, worm.lake, and their interacions

**6) model9** 21 1760.933

model9 <- glm(infected.yes.no ~ cop.lake + worm.lake + cop.lake*worm.lake + worm.fam, data = copepods, family = "binomial") #testing if worm fam had an effect on the prevalence of infection (not, it didn’t)

**7) model7** 65 1788.556

model7 <- glm(infected.yes.no ~ cop.lake + worm.lake + cop.lake*worm.lake + plate,

data=copepods,family="binomial") #testing if plate had an effect on the prevalence of infection: no, it didn’t

**8) model4** 4 2163.787

model4 <- glmer(infected.yes.no ~ worm.lake + (1|plate), data = copepods, family = "binomial") #testing for effect of worm.lake in prevalence of infection

**9) model5** 2 2171.093

model5 <- glmer(infected.yes.no ~ (1|plate), data = copepods, family ="binomial")

**#### Analyzing intensity of infection (i.e. number of tapeworms per infected copepod)**

Note: data is Poisson distributed

#Then using the following model:

**7) Analyzing results on intensity including those not infected (i.e. number of worms >= 0)**

> model10 = glm (numb.worm ~ cop.lake + worm.lake + cop.lake*worm.lake,

+ data=copepods,family="poisson")

> summary(model10)

Call:

glm(formula = numb.worm ~ cop.lake + worm.lake + cop.lake * worm.lake,

family = "poisson", data = copepods)

Deviance Residuals:

Min 1Q Median 3Q Max

-1.6042 -0.6794 -0.5164 0.1574 3.5375

Coefficients:

(Intercept) -1.8302 0.2425 -7.546 4.48e-14 ***

cop.lakeech 1.9931 0.2557 7.796 6.38e-15 ***

cop.lakegos 2.0824 0.2540 8.197 2.47e-16 ***

cop.lakelau -0.2422 0.3382 -0.716 0.47382

cop.lakerob 0.8747 0.3299 2.652 0.00801 **

worm.lakeech 0.3639 0.3263 1.115 0.26470

worm.lakegos 0.2461 0.3483 0.707 0.47984

cop.lakeech:worm.lakeech -0.5104 0.3480 -1.467 0.14248

cop.lakegos:worm.lakeech -0.5044 0.3440 -1.466 0.14260

cop.lakelau:worm.lakeech -0.1741 0.4578 -0.380 0.70367

cop.lakerob:worm.lakeech -0.3128 0.4640 -0.674 0.50015

cop.lakeech:worm.lakegos -0.5809 0.3719 -1.562 0.11827

cop.lakegos:worm.lakegos -0.6599 0.3685 -1.791 0.07336 .

cop.lakelau:worm.lakegos -0.5585 0.4983 -1.121 0.26241

cop.lakerob:worm.lakegos -0.8069 0.5314 -1.518 0.12892

---

Signif. codes: 0 ‘***’ 0.001 ‘**’ 0.01 ‘*’ 0.05 ‘.’ 0.1 ‘ ’ 1

(Dispersion parameter for poisson family taken to be 1)

Null deviance: 2107.7 on 1621 degrees of freedom

Residual deviance: 1498.2 on 1607 degrees of freedom

AIC: 2959.8

Number of Fisher Scoring iterations: 6

> anova(model10, test = "LRT")

Analysis of Deviance Table

Model: poisson, link: log

Response: numb.worm

Terms added sequentially (first to last)

Df Deviance Resid. Df Resid. Dev Pr(>Chi)

NULL 1621 2107.7

cop.lake 4 584.18 1617 1523.5 < 2.2e-16 ***

worm.lake 2 19.58 1615 1504.0 5.61e-05 ***

cop.lake:worm.lake 8 5.72 1607 1498.2 0.6781

---

Signif. codes: 0 ‘***’ 0.001 ‘**’ 0.01 ‘*’ 0.05 ‘.’ 0.1 ‘ ’ 1

**Results: It seems that copepod lake and worm lake have significant effects on intensity, but not on their interactions**

**8) What if I include a fixed variable in there, let’s say tapeworm family, and using “glmer” for GLMM**

> model11 <- glmer(numb.worm ~ cop.lake*worm.lake + (1|worm.fam),

+ data=copepods,family="poisson")

> summary(model11)

Generalized linear mixed model fit by maximum likelihood (Laplace Approximation) [glmerMod]

Family: poisson ( log )

Formula: numb.worm ~ cop.lake * worm.lake + (1 | worm.fam)

Data: copepods

AIC BIC logLik deviance df.resid

2961.7 3047.9 -1464.8 2929.7 1606

Scaled residuals:

Min 1Q Median 3Q Max

-1.1474 -0.4741 -0.3551 0.1667 7.4149

Random effects:

Groups Name Variance Std.Dev.

worm.fam (Intercept) 0.001736 0.04166

Number of obs: 1622, groups: worm.fam, 10

Fixed effects:

Estimate Std. Error z value Pr(>|z|)

(Intercept) -1.8330 0.2438 -7.517 5.61e-14 ***

cop.lakeech 1.9938 0.2557 7.798 6.27e-15 ***

cop.lakegos 2.0835 0.2541 8.201 2.39e-16 ***

cop.lakelau -0.2397 0.3383 -0.709 0.47863

cop.lakerob 0.8760 0.3299 2.655 0.00793 **

worm.lakeech 0.3672 0.3282 1.119 0.26322

worm.lakegos 0.2494 0.3501 0.712 0.47618

cop.lakeech:worm.lakeech -0.5132 0.3482 -1.474 0.14044

cop.lakegos:worm.lakeech -0.5069 0.3442 -1.473 0.14082

cop.lakelau:worm.lakeech -0.1784 0.4581 -0.389 0.69697

cop.lakerob:worm.lakeech -0.3201 0.4645 -0.689 0.49076

cop.lakeech:worm.lakegos -0.5813 0.3718 -1.563 0.11795

cop.lakegos:worm.lakegos -0.6605 0.3685 -1.792 0.07310 .

cop.lakelau:worm.lakegos -0.5613 0.4984 -1.126 0.26013

cop.lakerob:worm.lakegos -0.8078 0.5315 -1.520 0.12855

---

Signif. codes: 0 ‘***’ 0.001 ‘**’ 0.01 ‘*’ 0.05 ‘.’ 0.1 ‘ ’ 1

> AIC(model10,model11)

df AIC

model10 15 2959.820 #glm: numb.worm ~ worm.lake + cop.lake + worm.lake*cop.lake

model11 16 2961.659 #glmer: numb.worm ~ worm.lake*cop.lake + worm.fam [fixed var]

Summary: both models seem pretty similar

**9) what if I select only those copepods that got infected for the intensity analysis (as it should be)?**

preva = filter (copepods, numb.worm > 0) #using “filter” in “dplyer” R package to extract infected cops from dataset.

hist(preva$numb.worm, ylab = "# copepods")

#Data is still Poisson distributed.

model12 = glm (numb.worm ~ cop.lake + worm.lake + cop.lake*worm.lake,

data = preva,family="poisson")

Deviance Residuals:

Min 1Q Median 3Q Max

-0.7200 -0.4976 -0.1582 0.2137 2.0344

Coefficients:

Estimate Std. Error z value Pr(>|z|)

(Intercept) 0.060625 0.242536 0.250 0.8026

cop.lakeech 0.515466 0.255655 2.016 0.0438 *

cop.lakegos 0.582373 0.254043 2.292 0.0219 *

cop.lakelau -0.003466 0.338200 -0.010 0.9918

cop.lakerob 0.101894 0.329884 0.309 0.7574

worm.lakeech 0.093526 0.326255 0.287 0.7744

worm.lakegos 0.072907 0.348315 0.209 0.8342

cop.lakeech:worm.lakeech -0.251881 0.348006 -0.724 0.4692

cop.lakegos:worm.lakeech -0.198414 0.344046 -0.577 0.5641

cop.lakelau:worm.lakeech 0.003466 0.457840 0.008 0.9940

cop.lakerob:worm.lakeech -0.061889 0.463968 -0.133 0.8939

cop.lakeech:worm.lakegos -0.291160 0.368767 -0.790 0.4298

cop.lakegos:worm.lakegos -0.256372 0.368523 -0.696 0.4866

cop.lakelau:worm.lakegos 0.093078 0.493503 0.189 0.8504

cop.lakerob:worm.lakegos -0.117643 0.531446 -0.221 0.8248

---

Signif. codes: 0 ‘***’ 0.001 ‘**’ 0.01 ‘*’ 0.05 ‘.’ 0.1 ‘ ’ 1

(Dispersion parameter for poisson family taken to be 1)

Null deviance: 238.68 on 621 degrees of freedom

Residual deviance: 211.90 on 607 degrees of freedom

AIC: 1673.5

Number of Fisher Scoring iterations: 4

> anova(model12, test = "LRT")

Analysis of Deviance Table

Model: Poisson, link: log

Response: numb.worm

Terms added sequentially (first to last)

Df Deviance Resid. Df Resid. Dev Pr(>Chi)

NULL 621 238.68

cop.lake 4 21.5604 617 217.12 0.0002451 ***

worm.lake 2 3.3173 615 213.80 0.1903991

cop.lake:worm.lake 8 1.9074 607 211.90 0.9837214

---

Signif. codes: 0 ‘***’ 0.001 ‘**’ 0.01 ‘*’ 0.05 ‘.’ 0.1 ‘ ’ 1

Results: with only the infected copepods, it seems like now only the copepod lakes explains the results. We have the distinct hunch that it has to be either Echo Lake and/or Gosling lake’s copepods who are explaining most of these results.

**10) What if we include a GLMM model using worm fam as the fix variable (only for the infected copepods; this is for intensity):**

Generalized linear mixed model fit by maximum likelihood (Laplace Approximation)

model13 <- glmer(numb.worm ~ cop.lake*worm.lake + (1|worm.fam),

data = preva,family="poisson")

AIC BIC logLik deviance df.resid

1675.5 1746.4 -821.7 1643.5 606

Scaled residuals:

Min 1Q Median 3Q Max

-0.6541 -0.4636 -0.1543 0.2195 2.5118

Random effects:

Groups Name Variance Std.Dev.

worm.fam (Intercept) 0 0

Number of obs: 622, groups: worm.fam, 10

Fixed effects:

Estimate Std. Error z value Pr(>|z|)

(Intercept) 0.060625 0.242545 0.250 0.8026

cop.lakeech 0.515466 0.255665 2.016 0.0438 *

cop.lakegos 0.582373 0.254052 2.292 0.0219 *

cop.lakelau -0.003466 0.338206 -0.010 0.9918

cop.lakerob 0.101894 0.329891 0.309 0.7574

worm.lakeech 0.093526 0.326264 0.287 0.7744

worm.lakegos 0.072907 0.348328 0.209 0.8342

cop.lakeech:worm.lakeech -0.251881 0.348015 -0.724 0.4692

cop.lakegos:worm.lakeech -0.198414 0.344055 -0.577 0.5641

cop.lakelau:worm.lakeech 0.003466 0.457846 0.008 0.9940

cop.lakerob:worm.lakeech -0.061889 0.463976 -0.133 0.8939

cop.lakeech:worm.lakegos -0.291160 0.368781 -0.790 0.4298

cop.lakegos:worm.lakegos -0.256372 0.368537 -0.696 0.4866

cop.lakelau:worm.lakegos 0.093078 0.493512 0.189 0.8504

cop.lakerob:worm.lakegos -0.117643 0.531449 -0.221 0.8248

---

Signif. codes: 0 ‘***’ 0.001 ‘**’ 0.01 ‘*’ 0.05 ‘.’ 0.1 ‘ ’ 1

> AIC(model12,model13)

df AIC

model12 15 1673.494

model13 16 1675.494

Results: both models seem very similar

**11) Local adaption on intensity of infection levels (using only infected copepods for analyses):**

> summary(model16)

Call:

glm(formula = numb.worm ~ cop.lake + worm.lake + native, family = "poisson",

data = preva)

Deviance Residuals:

Min 1Q Median 3Q Max

-0.7056 -0.4897 -0.1383 0.1908 2.0068

Coefficients:

Estimate Std. Error z value Pr(>|z|)

(Intercept) 0.201136 0.143104 1.406 0.15987

cop.lakeech 0.354473 0.145691 2.433 0.01497 *

cop.lakegos 0.430396 0.144167 2.985 0.00283 **

cop.lakelau -0.003657 0.195147 -0.019 0.98505

cop.lakerob 0.013441 0.202257 0.066 0.94702

worm.lakeech -0.076399 0.078612 -0.972 0.33112

worm.lakegos -0.112841 0.087777 -1.286 0.19860

nativeTRUE -0.065931 0.081718 -0.807 0.41977

---

Signif. codes: 0 ‘***’ 0.001 ‘**’ 0.01 ‘*’ 0.05 ‘.’ 0.1 ‘ ’ 1

(Dispersion parameter for poisson family taken to be 1)

Null deviance: 238.68 on 621 degrees of freedom

Residual deviance: 213.15 on 614 degrees of freedom

AIC: 1660.7

> anova(model16, test = "LRT")

Analysis of Deviance Table

Model: Poisson, link: log

Response: numb.worm

Terms added sequentially (first to last)

Df Deviance Resid. Df Resid. Dev Pr(>Chi)

NULL 621 238.68

cop.lake 4 21.5604 617 217.12 **0.0002451 *****

worm.lake 2 3.3173 615 213.80 0.1903991

native 1 0.6538 614 213.15 0.4187510

---

Signif. codes: 0 ‘***’ 0.001 ‘**’ 0.01 ‘*’ 0.05 ‘.’ 0.1 ‘ ’ 1

Results: again, only copepod lake seems to account for the data

**The best models for intensity of infection (using only the data from infected copepods):**

Below are the models sorted from best to worst: (the first number after each model name is the degrees of freedom followed by the Akaike Information Criterion (AIC) value:

**Df AIC**

1. model 16 8 1660.748

glm (numb.worm ~ cop.lake + worm.lake + native, data = preva, family="poisson")

1. Model 12 15 1673.494

glm (numb.worm ~ cop.lake + worm.lake + cop.lake*worm.lake, data = preva, family="poisson")

1. Model 13 16 1675.494

glmer(numb.worm ~ cop.lake*worm.lake + (1|worm.fam), data = preva, family="poisson")
